# Plasma metabolomic characterization of premature ovarian insufficiency

**DOI:** 10.1186/s13048-022-01085-y

**Published:** 2023-01-05

**Authors:** Xing-Yu Zhou, Xin Li, Jun Zhang, Ying Li, Xiao-Min Wu, Yi-Zhen Yang, Xiao-Fei Zhang, Lin-Zi Ma, Yu-Dong Liu, Zhe Wang, Shi-Ling Chen

**Affiliations:** Center for Reproductive Medicine, Department of Gynecology and Obstetrics, Nanfang Hospital, Southern Medical University, No 1838 Guangzhou Northern Road, Guangzhou, 510515 People’s Republic of China

**Keywords:** Premature ovarian insufficiency, Metabolomics, Ovarian reserve, Arachidonoyl amides, Caffeine metabolism

## Abstract

**Background:**

Premature ovarian insufficiency (POI) patients are predisposed to metabolic disturbances, including in lipid metabolism and glucose metabolism, and metabolic disorders appear to be a prerequisite of the typical long-term complications of POI, such as cardiovascular diseases or osteoporosis. However, the metabolic changes underlying the development of POI and its subsequent complications are incompletely understood, and there are few studies characterizing the disturbed metabolome in POI patients. The aim of this study was to characterize the plasma metabolome in POI by using ultrahigh-performance liquid chromatography–mass spectrometry (UHPLC–MS/MS) metabolomics and to evaluate whether these disturbances identified in the plasma metabolome relate to ovarian reserve and have diagnostic value in POI.

**Methods:**

This observational study recruited 30 POI patients and 30 age- and body mass index (BMI)-matched controls in the Center for Reproductive Medicine, Department of Gynecology and Obstetrics, Nanfang Hospital, Southern Medical University, from January 2018 to October 2020. Fasting venous blood was collected at 9:00 am on days 2–4 of the menstrual cycle and centrifuged for analysis. An untargeted quantitative metabolomic analysis was performed using UHPLC–MS/MS.

**Results:**

Our study identified 48 upregulated and 21 downregulated positive metabolites, and 13 upregulated and 48 downregulated negative metabolites in the plasma of POI patients. The differentially regulated metabolites were involved in pathways such as caffeine metabolism and ubiquinone and other terpenoid-quinone biosynthesis. Six metabolites with an AUC value > 0.8, including arachidonoyl amide, 3-hydroxy-3-methylbutanoic acid, dihexyl nonanedioate, 18-HETE, cystine, and PG (16:0/18:1), were correlated with ovarian reserve and thus have the potential to be diagnostic biomarkers of POI.

**Conclusion:**

This UHPLC–MS/MS untargeted metabolomics study revealed differentially expressed metabolites in the plasma of patients with POI. The differential metabolites may not only be involved in the aetiology of POI but also contribute to its major complications. These findings offer a panoramic view of the plasma metabolite changes caused by POI, which may provide useful diagnostic and therapeutic clues for POI disease.

**Supplementary Information:**

The online version contains supplementary material available at 10.1186/s13048-022-01085-y.

## Introduction

Premature ovarian insufficiency (POI) refers to the exhaustion of ovarian reproductive and endocrine function before the age of 40 [1]. In POI, the most common symptoms are menstrual disturbance (amenorrhea or oligomenorrhea), hypoestrogenism, and infertility, and the most serious consequence is the conspicuously increased risk of cardiovascular diseases [2], osteoporosis [3], neurodegenerative disease [4], and type 2 diabetes mellitus [5], which are attributed to the lack of the protective effects of oestrogen. Although multiple factors, including genetic mutations, immune dysfunction, and iatrogenic factors such as chemotherapy, radiotherapy and ovarian surgery, have been reported to contribute to POI, the causes of most cases are still unclear [6]. Clinically, follicle stimulating hormone (FSH) > 25 mIU/ml is the only objective diagnostic parameter of POI [1], but FSH does not respond sensitively to diminished ovarian reserve [7]. Furthermore, although the symptoms caused by oestrogen deficiency can be improved by hormone replacement therapy (HRT), POI is an incurable condition. Considering the serious consequences of POI, it is essential to identify reliable biomarkers for diagnosing and predicting the disease course.

Metabolomics is a promising tool for the dynamic investigation of global metabolic responses to biological stimuli or diseases [8]. Compared to the scale of the genome, transcriptome and proteome, the metabolome is small, including only approximately 3000 low molecular weight end-products; hence, identification and quantification in metabolomics is fast and suitable for clinical application [9]. Recently, some metabolite features of the ovarian reserve decline process were identified via metabolomics. The anti-Müllerian hormone (AMH) level was regarded as the best biological measure of ovarian reserve, and a metabolomics study identified 14 serum metabolites, including phosphate, N-acetyl-D-glucosamine, and proline, that were associated with rapid AMH decline and reflected the progress of ovarian ageing [10]. Liang C et al. found that oxylipin metabolism disorders in follicular fluid were closely related to ovarian reserve function via targeted oxylipin metabolomics and identified 20-HDoHE, ± 5-iso PGF_2α_-VI, etc., as potential biomarkers for diminished ovarian reserve (DOR) [11], a part of which is usually considered the early stage of POI.

Although there are few metabolomic studies delineating the disturbed metabolome in POI patients, previous studies have suggested that abnormalities in lipid metabolism and glucose metabolism may be correlated with the pathophysiology of POI [12], and the risk of metabolic syndrome in POI patients was significantly higher than that in controls [13], indicating that there are metabolic disturbances in POI patients. Moreover, metabolic disorder appears to be an inherent component of the typical long-term complications of POI, such as cardiovascular diseases or osteoporosis, and the metabolic changes underlying the development of POI and its subsequent complications are incompletely understood. Therefore, the aim of this study was to characterize the plasma metabolome in POI by using ultrahigh-performance liquid chromatography–mass spectrometry (UHPLC–MS/MS) metabolomics and to evaluate whether these disturbances identified in the plasma metabolome relate to ovarian reserve and have diagnostic value in POI.

## Materials and methods

### Participants

This study included 60 participants recruited from the Center for Reproductive Medicine, Department of Gynecology and Obstetrics, Nanfang Hospital, Southern Medical University, from January 2018 to October 2020, consisting of 30 POI patients and 30 age- and body mass index (BMI)-matched controls. Permission was granted by the Ethics Committee of Nanfang Hospital of Southern Medical University (NFEC-2017-197), and written informed consent was obtained from all participants.

The inclusion criteria for POI were based on the guidelines produced by the European Society for Human Reproduction and Embryology (ESHRE) [1] and included (i) < 40 years of age, (ii) oligo/amenorrhea for at least 4 months, and (iii) a basal FSH level > 25 IU/L on two occasions > 4 weeks apart. The inclusion criteria for control women were as follows: (i) < 40 years of age, (ii) basal FSH < 10 IU/ml, (iii) bilateral antral follicle count (AFC) > 7, (iv) regular menstrual cycles occurring every 25–35 days, and (v) entered the in vitro fertilization/intracytoplasmic sperm injection (IVF/ICSI) program for male factor or tubal factor infertility.

Participants were excluded if they had (i) a history of other reproductive endocrine disorders, such as polycystic ovary syndrome (PCOS), hyperprolactinemia, endometriosis, and hypogonadotropic amenorrhea; (ii) a history of other endocrine disorders, such as hyperthyroidism, thyroiditis, and diabetes mellitus; (iii) a history of metabolic disturbance, such as BMI > 24 kg/m^2^, hyperlipidaemia, and hyperuricaemia; (iv) abnormal reproductive system anatomy or an abnormal karyotype; (v) a history of radiotherapy, chemotherapy and ovarian operation; (vi) a history of cardiovascular, neurological, psychiatric or other systemic diseases; or (vii) taken hormonal medications, such as oral contraceptives, hormone replacement therapy, insulin therapy, and thyroid medication, within the previous 3 months.

### Sample collection and pre-treatment

Fasting venous blood was collected in Ethylene Diamine Tetraacetic Acid (EDTA) anticoagulant tubes at 9:00 am on day 2–4 of the menstrual cycle. The blood samples were centrifuged at 2000 × rpm for 10 min at 4 °C and centrifuged again at 13,000 × rpm for 10 min at 4 °C within 1 h after collection to remove insoluble particles and cells. Then, the plasma was collected and kept frozen at − 80 °C until assessment.

During the pre-treatment, plasma samples were thawed at 4 °C, and 100 µL samples were resuspended in prechilled 80% methanol and 0.1% formic acid. Afterwards, the mixture was vortexed for 1 min, incubated on ice for 5 min and centrifuged at 15,000 g and 4 °C for 20 min. The supernatant was diluted to a final concentration containing 53% methanol in liquid chromatography–mass spectrometry (LC–MS)-grade water, subsequently transferred to a fresh Eppendorf tube and then centrifuged at 15,000 g and 4 °C for 20 min to collect the supernatant. Before UHPLC–MS/MS system analysis, equal volumes of each sample were mixed and used for quality control (QC).

### UHPLC–MS/MS analysis

UHPLC–MS/MS analyses were performed by Novogene Co., Ltd. (Beijing, China) using a Vanquish UHPLC system (Thermo Fisher, Bremen, Germany) coupled with an Orbitrap Q Exactive™ HF mass spectrometer (Thermo Fisher). UHPLC separation was performed on a Hypersil Goldcolumn (C18, 100 × 2.1 mm, 1.9 μm) using a 17-min linear gradient at a flow rate of 0.2 mL/min. Mobile phases A and B were 0.1% formic acid in water and methanol for positive polarity mode and 5 mM ammonium acetate, pH 9.0, and methanol for the negative polarity mode. The solvent gradient was set as follows: 2% B, 1.5 min; 2-100% B, 12.0 min; 100% B, 14.0 min; 100-2% B, 14.1 min; 2% B, 17 min. The Q Exactive™ HF mass spectrometer was operated in positive/negative polarity mode with a spray voltage of 3.2 kV, capillary temperature of 320 °C, sheath gas flow rate of 40 arb and aux gas flow rate of 10 arb.

### Data processing and metabolite identification

The raw data were processed in Compound Discoverer 3.1 (CD3.1, Thermo Fisher) for peak alignment, peak picking, and quantitation for each metabolite. Peak intensities normalized by total spectral intensity were used to predict the molecular formula based on additive ions, molecular ion peaks and fragment ions. Databases including mzCloud (https://www.mzcloud.org/), mzVault and MassList were used to obtain accurate qualitative and relative quantitative results matched with peaks.

### Data analysis

SPSS version 24.0 (IBM, Armonk, NY, USA) was used for statistical evaluation of clinical characteristics. Continuous variables with a normal distribution were compared with Student’s t test and are presented as the mean ± SD. Otherwise, variables were compared with the Mann–Whitney U test and are presented as the median (interquartile range, IQR). Spearman rank correlation coefficient analysis was used for correlation analyses.

The Kyoto Encyclopedia of Genes and Genomes (KEGG) database (https://www.genome.jp/kegg/pathway.html), HMDB database (https://hmdb.ca/metabolites) and LIPID MAPS database (http://www.lipidmaps.org/) were used to annotate metabolites. The statistical software R (R version R-3.4.3), Python (Python 2.7.6 version) and CentOS (CentOS release 6.6) were applied to statistical analyses of metabolites. The relative quantitative results generated from the data processing step were log2 transformed, followed by univariate scaling. Then, Student’s t-test was applied to calculate the statistical significance of metabolites between two groups and a *P*-value < 0.05 was considered as statistically significant. Principal component analysis (PCA) and partial least squares discriminant analysis (PLS-DA) were performed with metaX. PCA was used to investigate the clustering trends of the samples and QCs, and PLS-DA was used to compare the classification between two groups. In PLS-DA, the variable importance in the projection (VIP) of the first principal component was calculated to evaluate the metabolite contributions to the model. Metabolites with a VIP value > 1, *P* value < 0.05 and fold change (FC) ≥ 2 or ≤ 0.5 were considered significantly different. The KEGG database was used to annotate the functions of the metabolites and metabolic pathways. Receiver operating characteristic (ROC) curves were created by cor.mtest() in metaX, an R package for metabolomic data analysis [14]. A random forest model was built via Python for performing cross validation. Specifically, numpy and pandas were used for data pre-treatment, sklearn was used for modelling, and matplotlib.pyplot was used for drawing.

## Results

### Participant characteristics

The clinical characteristics of the POI patients and controls are listed in Table 1. There was no significant difference between the POI and control groups in terms of age, BMI, or serum progesterone level (*P* > 0.05). Basal FSH and LH levels were significantly higher, and oestradiol levels were significantly lower, in POI patients than in controls (*P* < 0.05). The median (IQR) of the duration of POI was 4.00 (2.00-6.75) years, ranging from 0.25 to 12 year.

**Table 1:** Clinical characteristics of patients with premature ovarian insufficiency and controls

Variable	Control	POI	*P*
n	30	30	-
Age (year)	30.80 ± 3.15^a^	28.67 ± 4.95^a^	0.052
BMI	21.38 ± 1.54^a^	21.12 ± 3.56^a^	0.728
AMH (ng/mL)	2.73 (1.72, 3.62)^b^	0.01 (0.01, 0.07)b	< 0.001
AFC	14 (11, 17.5)b	0 (0, 1)b	< 0.001
Basal FSH (mIU/mL)	6.39 (5.91, 7.62)	92.35 (62.80, 105.40)^b^	< 0.001
Basal LH (mIU/mL)	5.08 (4.01, 6.68)b	39.42 (33.01, 61.55)^b^	< 0.001
Basal oestradiol (pg/mL)	37.69 (28.53, 50.57)b	15.90 (5.00, 27.10)^b^	< 0.001
Basal progesterone (ng/mL)	0.34 (0.14, 0.50)b	0.37 (0.20, 1.0)^b^	0.398

^a^Shown as mean ± SD

^b^Shown as median (interquartile range)

### Metabolite overview

**Fig. 1:**
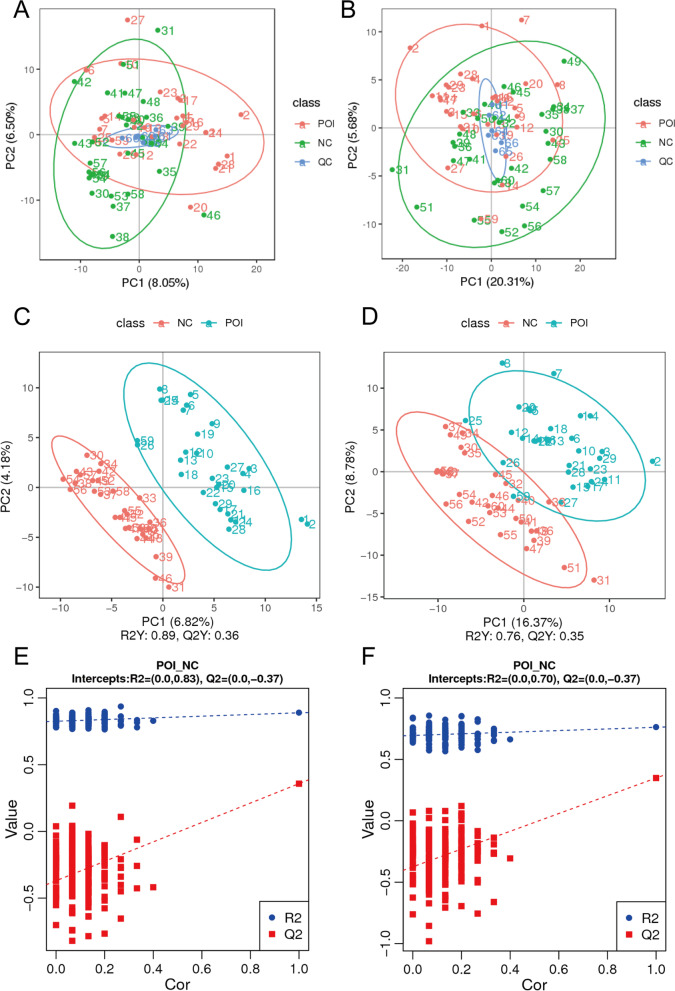
PCA score plots, PLS-DA score plots, and PLS-DA model permutation test derived from metabolomics profiles comparing the POI and control groups. **A** PCA score plot with QC samples in positive polarity mode. **B** PCA score plot with QC samples in negative polarity mode. **C** PLS-DA score plot in positive polarity mode. **D** PLS-DA score plot in negative polarity mode. **E** PLS-DA model permutation test in positive polarity mode. **F** PLS-DA model permutation test in negative polarity mode

The Pearson correlation coefficient between QC samples was calculated based on the relative quantitative value of the metabolites. The Pearson correlation coefficient was between 0.983 and 0.993 in positive polarity mode (Supplementary Fig. 1A) and between 0.985 and 0.992 in negative polarity mode (Supplementary Fig. 1B); the high correlation among QC samples indicated that the detection process was stable and data reliability was high. In the PCA plot (Fig. 1A-B), QC samples were clustered tightly near the coordinate origin, suggesting the stability of the UHPLC–MS/MS system. However, no clear separation was observed between the POI group and the control group in the PCA plot. In the PLS-DA analysis model, R2Y (R2) represents the probability interpretation of matrices, and Q2Y (Q2) is calculated by cross validation to evaluate the predictive power of PLS-DA model [15]. The closer the value of R2 and Q2 are to 1, the better the PLS-DA model is. In our analysis, the values of R2 = 0.89 and Q2 = 0.36 in positive polarity mode (Fig. 1C) and R2 = 0.76 and Q2 = 0.35 (Fig. 1D) in negative polarity mode indicated the good reliability, but suboptimal predictive power of this model. However, in view of the high heterogeneity of the human samples and the aetiological heterogeneity of POI, we thought there Q2 values of more than 0.3 were acceptable. In addition, sorting validation (Fig. 1E F) was used to assess whether the PLS-DA model was overfitted. When the R2 value was greater than the Q2 value and the intercept between the regression line of Q2 and Y-axis was less than 0, the model could be identified as being no overfitted. Figure 1E F indicates the lack of overfitting in both the positive polarity mode and negative mode.

### Identification of significantly differentially abundant metabolites

**Fig. 2:**
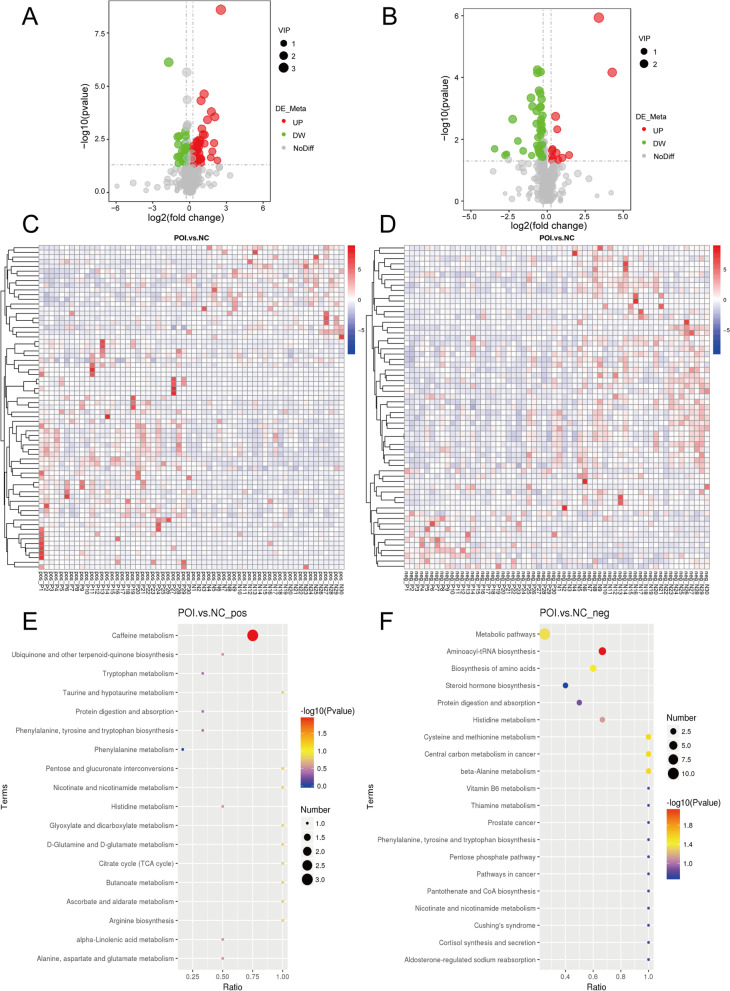
Identification of the differential metabolomic profiles between the POI and control groups. **A** Volcano plot visualizing the differentially regulated metabolites in POI compared to the control in positive polarity mode (fold change≥ 2 or ≤ 0.5, *p* < 0.05). **B** Volcano plot in negative polarity mode (fold change ≥ 2 or ≤ 0.5, *p* < 0.05). **C** Heatmap clustering presenting differentially regulated metabolites in positive polarity mode. **D** Heatmap clustering presenting differentially regulated metabolites in negative polarity mode. **E** KEGG pathway analysis for identifying the metabolic pathways associated with the differentially abundant metabolites between the indicated groups in positive polarity mode. **F** KEGG pathway analysis for negative polarity mode

A total of 576 positive metabolites and 385 negative metabolites were detected in the UHPLC–MS/MS analysis. These metabolites were mainly involved in lipid metabolism, amino acid metabolism, nucleotide metabolism, etc. (Supplementary Fig. 2A-B) as annotated by KEGG, and glycerophospholipids and fatty acyls were the most important types of lipids (Supplementary Fig. 2C-D) as annotated by LIPID MAPS. Of these, 48 upregulated and 21 downregulated positive metabolites (Fig. 2A); and 13 upregulated and 48 downregulated negative metabolites (Fig. 2B) were identified to be significantly differentially expressed in the POI group compared with the control group. Heatmaps with hierarchical clustering analysis (Fig. 2A-B) and volcano plots (Fig. 2C-D) provided an overview of significantly different metabolites.

The characteristics of the top 20 differentially abundant metabolites in positive/negative polarity mode are shown in Table 2. KEGG pathway analysis was conducted to explore the potential pathways associated with those differentially abundant metabolites. Figure 2E-F shows the most significant metabolic pathways attributed to POI, including caffeine metabolism, ubiquinone and other terpenoid-quinone biosynthesis, and tryptophan metabolism in positive polarity mode and metabolic pathways, aminoacyl-tRNA biosynthesis, and biosynthesis of amino acids in negative polarity mode.

**Table 2:** Characterization of the top 20 differential metabolites in positive/negative polarity mode

Compound_ID	Name	Formula	Fold change	log2FC	VIP	*P* value	Up/Down
Com_19056_pos	Arachidonoyl amide	C_20_H_33_NO	5.852882	2.549147	3.311601	2.54E-09	up
Com_6551_pos	3-Hydroxy-3-methylbutanoic acid	C_5_H_10_O_3_	0.305806	-1.70931	2.73131	7.48E-07	down
Com_2458_pos	Dihexyl nonanedioate	C_21_H_40_O_4_	2.270675	1.183121	2.448546	2.34E-05	up
Com_10406_pos	18-HETE	C_20_H_32_O_3_	1.904571	0.929466	2.283455	4.76E-05	up
Com_32725_pos	Lypressin Acetate	C_46_H_65_N_13_O_12_S_2_	3.42008	1.77403	2.260713	0.000158	up
Com_10863_pos	7-(2-hydroxypropan-2-yl)-1,4a-dimethyl-decahydronaphthalen-1-ol	C_15_H_28_O_2_	4.176609	2.062332	2.209279	0.000276	up
Com_7281_pos	Nordiazepam	C_15_H_11_ClN_2_O	2.746943	1.457827	2.086147	0.00038	up
Com_19603_pos	Wogonin	C_16_H_12_O_5_	1.998795	0.999131	1.961295	0.001005	up
Com_26_pos	DL-Tryptophan	C_11_H_12_N_2_O_2_	0.788134	-0.34349	1.850143	0.00134	down
Com_5033_pos	N-Desmethyltramadol	C_15_H_23_NO_2_	2.134642	1.093994	1.848938	0.001829	up
Com_8626_pos	PC (18:1e/14:1)	C_40_H_78_NO_7_P	2.331238	1.221096	1.827355	0.001932	up
Com_416_pos	2-Hydroxycinnamic acid	C_9_H_8_O_3_	0.802959	-0.3166	1.815953	0.002184	down
Com_1594_pos	Theophylline	C_7_H_8_N_4_O_2_	0.551395	-0.85884	1.826479	0.002225	down
Com_7482_pos	Vanillin	C_8_H_8_O_3_	0.507377	-0.97887	1.877203	0.002407	down
Com_6942_pos	2-(2-oxo-2-{[2-(2-oxo-1-imidazolidinyl)ethyl]amino}ethoxy)acetic acid	C_9_H_15_N_3_O_5_	1.703716	0.768685	1.701999	0.003268	up
Com_27637_pos	Lenalidomide	C_13_H_13_N_3_O_3_	1.834114	0.875084	1.704982	0.003655	up
Com_2551_pos	Anacardic acid	C_22_H_36_O_3_	1.506996	0.591675	1.811478	0.003867	up
Com_1910_pos	Ethyl chrysanthemumate	C_12_H_20_O_2_	1.551453	0.63362	1.707027	0.004293	up
Com_6799_pos	6-Methoxy-2-naphthoic acid	C_12_H_10_O_3_	3.988188	1.995733	1.687129	0.004703	up
Com_11318_pos	(2 S)-2-(2-hydroxypropan-2-yl)-2 H,3 H,7 H-furo[3,2-g]chromen-7-one	C_14_H_14_O_4_	0.77809	-0.36199	1.76194	0.004857	down
Com_4006_neg	GM3 d36:1; [M-H]-	C_59_H_108_N_2_O_21_	10.5212	3.395227	2.89096	1.16E-06	up
Com_5032_neg	Cystine	C_6_H_12_N_2_O_4_S_2_	0.643636	-0.63568	1.87981	5.61E-05	down
Com_2157_neg	L-Aspartic acid	C_4_H_7_NO_4_	0.739742	-0.43491	1.854329	6.56E-05	down
Com_2414_neg	PG (16:0/18:1)	C_40_H_77_O_10_P	19.33564	4.273191	2.267279	6.84E-05	up
Com_1106_neg	LPA 18:0	C_21_H_43_O_7_P	0.646888	-0.62841	1.842277	7.03E-05	down
Com_2079_neg	Hexanoic acid	C_6_H_12_O_2_	0.693318	-0.52841	1.714559	0.000266	down
Com_2027_neg	L-Threonic acid	C_4_H_8_O_5_	0.731709	-0.45066	1.691488	0.000323	down
Com_8027_neg	7-Hydroxy-3,4-dihydrocarbostyril	C_9_H_9_NO_2_	0.479844	-1.05936	1.818085	0.000456	down
Com_282_neg	Uric acid	C_5_H_4_N_4_O_3_	0.780041	-0.35838	1.631659	0.000501	down
Com_5948_neg	Daidzein	C_15_H_10_O_4_	0.513087	-0.96272	1.670357	0.000862	down
Com_6265_neg	Undecanoic acid	C_11_H_22_O_2_	0.744988	-0.42471	1.638692	0.000888	down
Com_8167_neg	N-Acetyl-aspartic acid	C_6_H_9_NO_5_	0.718234	-0.47747	1.594997	0.000939	down
Com_1957_neg	5-amino-1-phenyl-1 H-pyrazole-4-carbonitrile	C_10_H_8_N_4_	0.619616	-0.69055	1.526146	0.001222	down
Com_9084_neg	(5ξ,9ξ)-17-Hydroxykaur-15-en-19-oic acid	C_20_H_30_O_3_	0.812237	-0.30003	1.547688	0.001713	down
Com_2039_neg	Lignoceric Acid	C_24_H_48_O_2_	1.472289	0.55806	1.915449	0.00181	up
Com_3369_neg	PC (18:0e/18:2)	C_44_H_86_NO_7_P	0.208414	-2.26248	1.815235	0.002232	down
Com_3826_neg	Methionine	C_5_H_11_NO_2_S	0.758644	-0.39851	1.53752	0.002317	down
Com_518_neg	LPE 18:0	C_23_H_48_NO_7_P	0.749874	-0.41528	1.482862	0.002971	down
Com_819_neg	LPC 18:2	C_26_H_50_NO_7_P	0.61738	-0.69577	1.46772	0.003112	down
Com_1334_neg	DL-β-Leucine	C_6_H_13_NO_2_	0.782392	-0.35404	1.462225	0.003406	down

### Plasma metabolites with potential diagnostic value in POI

**Fig. 3:**
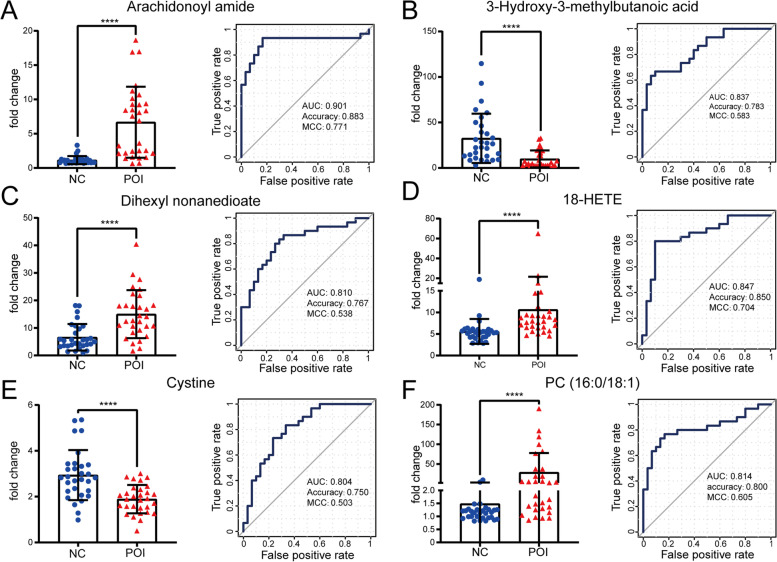
Identification of potential metabolite biomarkers of POI. **A** Comparison and ROC analysis for arachidonoyl amide. **B** 3-Hydroxy-3-methylbutanoic acid. **C** Dihexyl nonanedioate. **D** 18-HETE. **E** Cystine. **F** PG (16:0/18:1). ^****^ *p* < 0.0001

To identify metabolites that have the potential to be biomarkers for POI, ROC curves were built, and the area under the ROC curve (AUC), accuracy, and Matthew’s correlation coefficient (MCC) were calculated. Metabolites with an AUC value > 0.8 are listed in Fig. 3. The AUC value of arachidonoyl amide (Fig. 3A) was 0.901 (95% confidence interval [CI]: 0.810 to 0.993, sensitivity: 93.33%, specificity: 83.33%, *P* < 0.0001). The value was 0.837 (95% [CI]: 0.738 to 0.935, sensitivity: 66.67%, specificity: 90.00%, *P* < 0.0001) for 3-hydroxy-3-methylbutanoic acid (3-OH3MB) (Fig. 3B); 0.810 (95% [CI]: 0.699 to 0.921, sensitivity: 86.67%, specificity: 66.67%, *P* < 0.0001) for dihexyl nonanedioate (Fig. 3C); 0.847 (95% [CI]: 0.744 to 0.949, sensitivity: 80.00%, specificity: 90.00%, *P* < 0.0001) for 18-hydroxyeicosatetraenoic acid (HETE) (Fig. 3D); 0.804 (95% [CI]: 0.694 to 0.915, sensitivity: 73.33%, specificity: 76.67%, *P* < 0.0001) for cystine (Fig. 3E); and 0.814 (95% [CI]: 0.701 to 0.928, sensitivity: 76.67%, specificity: 83.33%, *P* < 0.0001) for PG (16:0/18:1) (Fig. 3F). Among them, arachidonoyl amide and 18-HETE achieved an accuracy value more than 0.850 and a MCC more than 0.700, indicating good performance of them.

Furthermore, we analysed the correlation between the aforementioned metabolites and age, AMH, AFC, and FSH, which are recognized clinical indicators of ovarian reserve. Spearman correlation analysis, shown in Table 3, revealed that the concentrations of arachidonoyl amide, dihexyl nonanedioate, 18- HETE, and PG (16:0/18:1) were positively correlated with basal FSH levels but negatively correlated with AMH and AFC. The concentrations of 3OH3MB and cystine were found to have a positive correlation with AMH and AFC but a negative correlation with basal FSH levels. No relationship between any of the six metabolites and age was observed in the Spearman correlation analysis.

**Table 3:** Spearman correlation analysis between the concentration of metabolites and ovarian reserve

	Arachidonoyl amide	3-Hydroxy-3-methylbutanoic acid	Dihexyl nonanedioate	18-HETE	Cystine	PG (16:0/18:1)
Age	-0.163	0.143	-0.076	-0.163	0.125	0.073
Basal FSH	0.551^**^	-0.507^**^	0.498^**^	0.523^**^	-0.474^**^	0.403^**^
AMH	-0.651^**^	0.494^**^	-0.413^*^	-0.502^**^	0.582^**^	-0.433^**^
AFC	-0.547^**^	0.453^**^	-0.475^**^	-0.451^**^	0.445^**^	-0.568^**^

*P < 0.05

**P < 0.01

### A random forest model

**Fig. 4:**
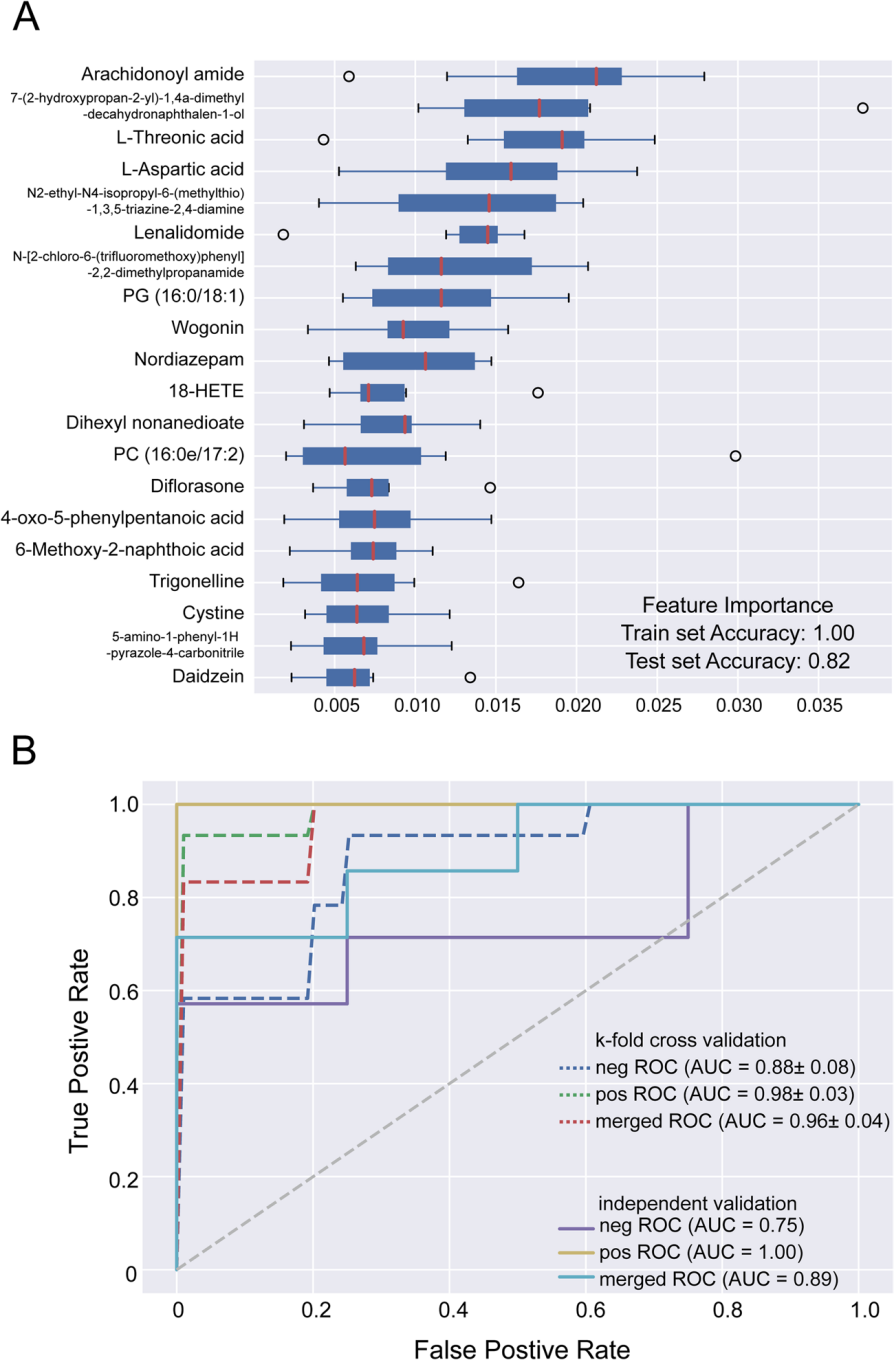
A random forest model for cross validation. **A** Box plots with the top 20 metabolites with high contribution in this random forest model. **B** ROC curve in the K-fold validation and independent validation

To obtain a system with better discriminatory power, a random forest model was built. As shown in Fig. 4A, the top 20 metabolites with high contributions were included in this random forest model. The ROC curve is shown in Fig. 4B: the merged AUC could reach 0.96 in the K-fold validation, and the merged AUC was 0.89 in the independent validation. The out-of-bag (oob) error was 0.244, 0.073, and 0.122 in negative train, positive train and merge train, respectively. Both accuracy and MCC was 1 in the three trains, which indicated the good predictive value of this random forest model.

## Discussion

Our study investigated the metabolomic features in the plasma of patients with POI via UHPLC–MS/MS analysis and demonstrated the alteration of 130 metabolites involved in pathways such as caffeine metabolism, ubiquinone biosynthesis and other terpenoid-quinone biosynthesis. Arachidonoyl amide, 3-3OH3MB, dihexyl nonanedioate, 18-HETE, cystine, and PG (16:0/18:1) were found to have the potential to be diagnostic biomarkers of POI.

In this study, we found that serum concentrations of caffeine metabolites such as theophylline, theobromine, and paraxanthine were decreased in patients with POI. Theophylline, a nonselective adenosine receptor antagonist and competitive nonselective phosphodiesterase inhibitor, has been found to have a negative effect on ovulation, oocyte maturation, and pregnancy in mammals [16], but there is no evidence to date indicating that theobromine or paraxanthine affect ovarian function. A previous study supported the hypothesis that coffee consumption may be helpful for reducing testosterone and improving menstrual cycle function in healthy premenopausal women [17]. However, other studies suggested that the relationship between drinking coffee and the age of menopause or ovarian reserve was ambiguous [18–20]. Although we did not collect the coffee intake in participants prospectively, the results in this study still provide some preliminary evidence that the appropriate drinking of coffee might be a protective factor in POI.

Here, we demonstrated that plasma arachidonoyl amide levels were significantly increased in patients with POI, with an AUC value of 0.901. Arachidonoyl amides act as ligands of endocannabinoid receptors [21], and anandamide (AEA) and 2-arachidonoyl glycerol (2-AG) are members of the arachidonoyl amide subfamily. Intriguingly, a recent plasma metabolomic study also found that plasma 2-AG levels were significantly increased in POI patients compared with both control women and PCOS patients [22]. More importantly, the endocannabinoid system has long been implicated in reproductive system metabolism. Both El-Talatini MR et al. and Cui N et al. revealed the same dynamic change in the plasma AEA level, namely, an increase in the follicular phase, peak at the time of ovulation, and minimum in the luteal phase [23, 24]. The similar trends of AEA levels and oestradiol and gonadotrophin levels during the menstrual cycle suggested that sex hormones are involved in the regulation of AEA levels. Franchi A further confirmed that anandamide-metabolizing enzymes were regulated by ovarian hormones and that AEA may compromise pregnancy outcome [25]. Conversely, the endocannabinoid system also plays a modulating role in the regulation of oestradiol synthesis, follicle maturation, oviductal embryo transport, implantation and placentation in females and participates in spermatogenesis in males [26–28]. Moreover, endocannabinoids can activate lipoprotein lipase, promote adipogenesis and fat deposition, and cause insulin resistance in adipose tissue and skeletal muscle [29]. Studies have shown that blocking the cannabinoid 1 (CB1) receptor leads to fat loss, reverses the overproduction of leptin and insulin, and has a protective effect on hyperinsulinemia and beta cell dysfunction [30]. Therefore, an increase in arachidonoyl amide levels may be involved in the pathogenesis of POI and the subsequent disorder of glucose and lipid metabolism. Reducing the level of endocannabinoids or blocking the CB1 receptor in patients with POI may improve clinical symptoms and prevent long-term complications.

18-HETE is a kind of oxylipin produced via C18 monohydroxylation of arachidonic acid catalysed by cytochrome P450 (CYP) oxygenases [31]. Oxylipins are oxygenated polyunsaturated fatty acids that act as bioactive lipids to regulate inflammation, immunity, and endocrine processes and are implicated in a variety of chronic inflammatory diseases, including cardiovascular disease, diabetes, and Alzheimer’s disease [32–34]. Recently, Liang C et al. revealed that the concentrations of 15 oxylipins related to the arachidonic acid metabolic pathway were decreased in the follicular fluid (FF) of DOR patients, and 8 of them were negatively correlated with FSH and positively correlated with AFC [11]. FF is a mixture of serum diffused from capillaries and the secretions of peripheral granulosa cells; thus, the composition of FF is close to that of plasma and represents the follicular microenvironment [35]. However, it is difficult to obtain FF from POI patients, and patient plasma was collected in this study. Our finding that the plasma levels of 18-HETE were increased in POI patients conflicts with the report from Liang C et al [11]. Their conclusion was also in contrast to the current understanding and our previous finding that excessive oxidative stress promotes the development of POI and DOR [36, 37]. They explained that the use of double-cavity needles and technical limitations of oxidized lipid metabolomics may account for this discrepancy. Given that patients’ plasma reflects the condition of the whole body and that 18-HETE reflects the oxidized lipids accumulated in the plasma, we speculated that POI patients have oxidative stress disorder, although thus far, the causal relationship between the two is unclear. On the one hand, evidence suggested that various ROS triggers could impair ovarian function and that some antioxidants, such as melatonin and resveratrol, could ameliorate excess ROS-induced damage, indicating that excessive oxidative stress may result in the incidence of POI [38]. On the other hand, oestrogen deficiency as a result of POI is associated with increased oxidative stress, inducing cardiac dysfunction and osteoporosis [39, 40]. To determine the causality between the metabolomics changes and POI, further prospective cohort studies are needed.

POI patients often suffer from an unfavourable lipid profile, specifically, hyperlipidaemia [41], higher total cholesterol and low-density lipoprotein [42], which is closely associated with higher cardiovascular risk. The mechanism of this abnormal lipid metabolism could be a lack of oestrogen leading to dysregulation of nuclear receptors, immune senescence, and oxidative stress in the liver [43]. We revealed that glycerophospholipids and fatty acyls were the most relevant lipid metabolism pathways in POI, in which PG (16:0/18:1) was positively correlated with basal FSH levels and negatively correlated with AMH and AFC, with a high ROC value of 0.814. Glycerophospholipids are important components of cell membranes and play a role in signal transmission, cell proliferation and other important physiological functions [44]. Aberrant glycerophospholipid metabolism is associated with osteoporosis [45], spontaneous abortion [46], and even dysfunction of granulosa cells [47]. Free fatty acids (FFAs) are important energy sources for the body. In some conditions, the disturbance of fatty acid homeostasis promotes the increased release of FFAs and triglycerides stored in tissues such as the liver, skeletal muscles, cardiac muscle, pancreas, kidney and brain, causing direct harmful effects; this is known as lipotoxicity [48]. Dysregulation of FFA metabolism may contribute to the risk of type 2 diabetes and cardiovascular diseases in POI patients due to the key roles of dysregulation of FFA metabolism in insulin resistance and dyslipidaemia [49].

Cystine is a major resource for the synthesis of glutathione, an essential antioxidative molecule in vivo; therefore, ingestion of cystine was shown to inhibit excess inflammation and oxidative stress after strenuous exercise or surgery [50–52]. In contrast, cystine deprivation was implicated in ferroptosis, a novel iron-dependent form of regulated cell death that is driven by excessive oxidative damage to lipids [53]. Although the relationship between ferroptosis and POI has not been clarified, the accumulated evidence indicates that ferroptosis contributes to the major complications of POI, including cardiovascular disease [54], neurodegenerative disease [55], and osteoporosis [56]. According to a recent report published in *Science*, cystine also acts as a key regulator in diet-regulated growth suppression programs, such as autophagy [57], which is critical for ovarian function and participates in the pathogenesis of POI [58].

Although the present study provides new insights into the metabolic pathophysiology of POI, it still has several limitations. First, there were no strict long-term dietary or exercise restrictions for participants. Fasting for one night may not be sufficient to eliminate the effects of diet. Second, we collected only plasma for the metabolomic analysis; therefore, other samples, such as urine, should be further analysed to validate our results. Third, all of the participants in this study were in overt stages of POI disease, meaning that their ovarian function was almost completely exhausted. The metabolite profile in late stages of POI may not represent the dynamic metabolomic changes that occur throughout the entire progression of the disease. On the one hand, the diagnostic criteria of occult and biochemical POI are not agreed upon; on the other hand, it is difficult to distinguish occult and biochemical POI from DOR. Therefore, we believe that this metabolomic study of overt POI will provide helpful information for the development of diagnostic criteria for occult and biochemical POI. Finally, the control group in this study is not ideal for studying plasma metabolic profiles in POI patients. IVF/ICSI treatment or infertility caused by male factors or tubal factors may result in altered metabolomic profiles. In the future, further targeted metabolome analysis in larger cohorts is highly recommended to validate the differential metabolites found in this study.

## Conclusion

This UHPLC–MS/MS untargeted metabolomics analysis revealed 130 differentially expressed metabolites in the plasma of patients with POI. Six metabolites related to ovarian reserve, namely, arachidonoyl amide, 3OH3MB, dihexyl nonanedioate, 18-HETE, cystine, and PG (16:0/18:1), yield good diagnostic performance and have the potential to be effective biomarkers for POI. The differentially abundant metabolites may not only be involved in the aetiology of POI but also contribute to its major complications. The findings offer a panoramic view of the plasma metabolite changes caused by POI, which may provide useful information for the diagnosis and treatment of POI.

## Supplementary Information

**Additional file 1: ** **Supplementary Fig 1.** Pearson correlation coefficient between QC samples. (A) Positive polarity mode. (B) Negative polarity mode. **Supplementary Fig 2.** KEGG and LIPID MAPS database annotation of metabolites detected by UHPLC–MS/MS analysis. (A) KEGG pathway annotation in positive polarity mode. (B) KEGG pathway annotation in negative polarity mode. (C) LIPID MAPS annotation in positive polarity mode. (D) LIPID MAPS annotation in negative polarity mode.

## Data Availability

The datasets used and/or analysed during the current study are available from the corresponding author on reasonable request.
