# Supplementary material for: Plasma metabolomic characterization of premature ovarian insufficiency
Source: J Ovarian Res. 2023 Jan 5;16:2. doi: 10.1186/s13048-022-01085-y (PMC9814329; doi:10.1186/s13048-022-01085-y)
Supplement: Supplementary file 1 — Additional file 1: Supplementary Fig 1. Pearson correlation coefficient between QC samples. (A) Positive polarity mode. (B) Negative polarity mode. Supplementary Fig 2. KEGG and LIPID MAPS database annotation of metabolites detected by UHPLC–MS/MS analysis. (A) KEGG pathway annotation in positive polarity mode. (B) KEGG pathway annotation in negative polarity mode. (C) LIPID MAPS annotation in positive polarity mode. (D) LIPID MAPS annotation in negative polarity mode. [file 13048_2022_1085_MOESM1_ESM.docx]

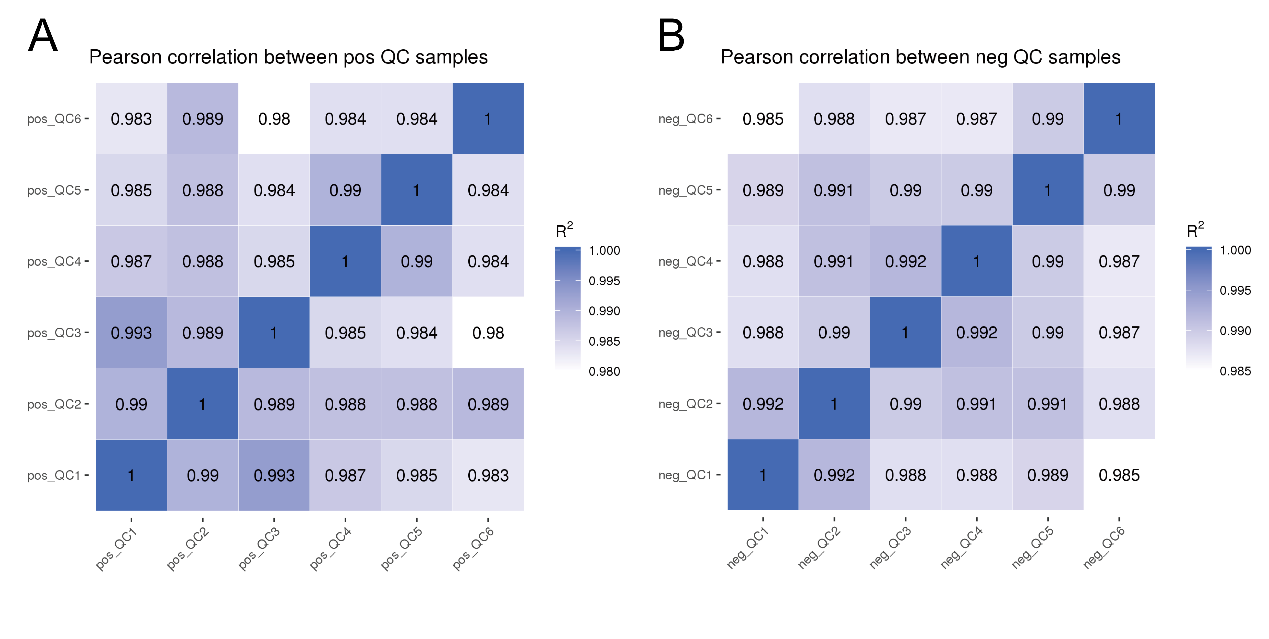


**Supplementary Fig 1 Pearson correlation coefficient between QC samples.** (A) Positive polarity mode. (B) Negative polarity mode.


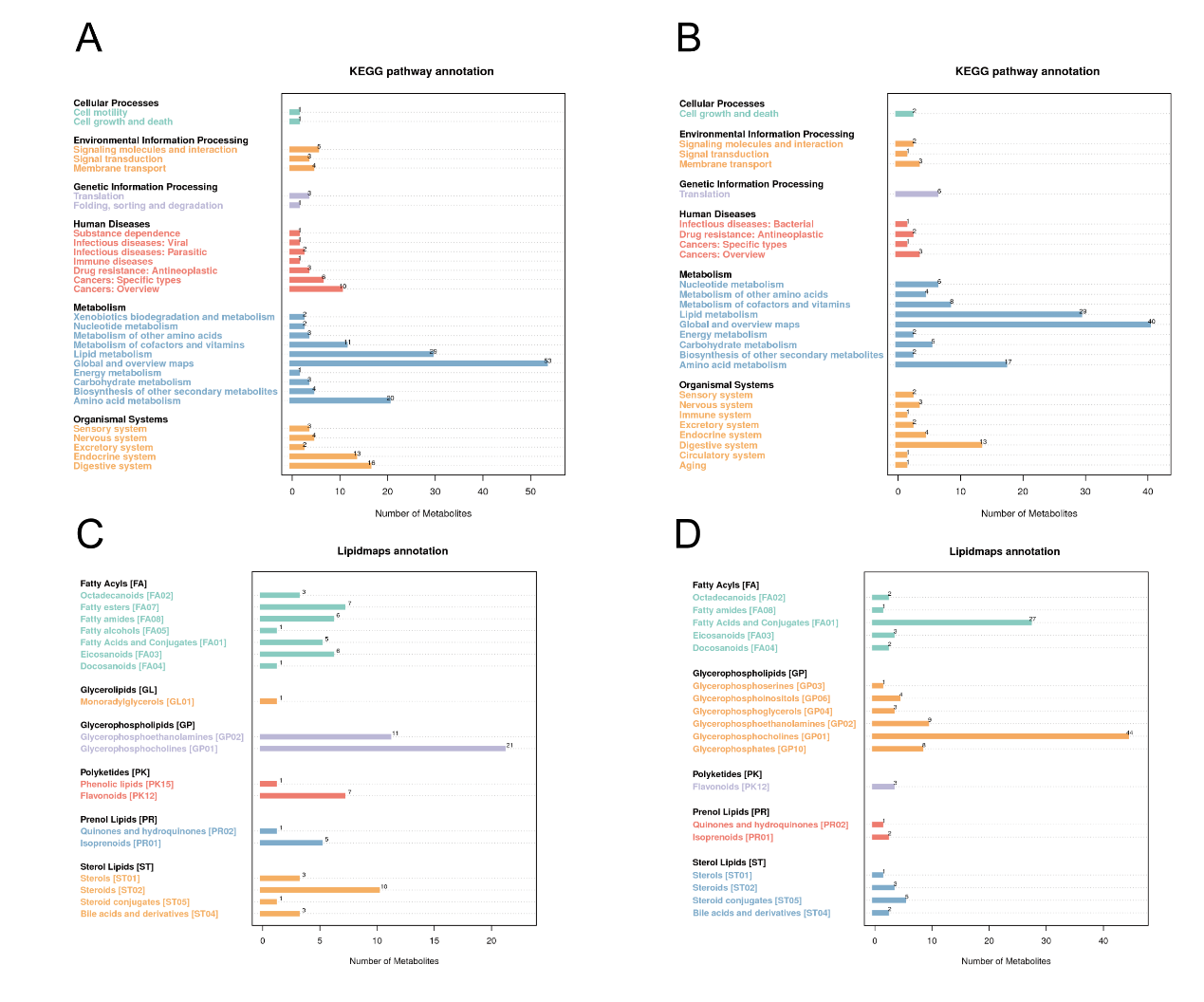


**Supplementary Fig 2 KEGG and LIPID MAPS database annotation of metabolites detected by UHPLC–MS/MS analysis.** (A) KEGG pathway annotation in positive polarity mode. (B) KEGG pathway annotation in negative polarity mode. (C) LIPID MAPS annotation in positive polarity mode. (D) LIPID MAPS annotation in negative polarity mode.
